# Myosin II activity dependent and independent vinculin recruitment to the sites of E-cadherin-mediated cell-cell adhesion

**DOI:** 10.1186/1471-2121-12-48

**Published:** 2011-11-03

**Authors:** Grant M Sumida, Tyler M Tomita, Wenting Shih, Soichiro Yamada

**Affiliations:** 1 Department of Biomedical Engineering, University of California, Davis Davis, CA 95616 USA

## Abstract

**Background:**

Maintaining proper adhesion between neighboring cells depends on the ability of cells to mechanically respond to tension at cell-cell junctions through the actin cytoskeleton. Thus, identifying the molecules involved in responding to cell tension would provide insight into the maintenance, regulation, and breakdown of cell-cell junctions during various biological processes. Vinculin, an actin-binding protein that associates with the cadherin complex, is recruited to cell-cell contacts under increased tension in a myosin II-dependent manner. However, the precise role of vinculin at force-bearing cell-cell junctions and how myosin II activity alters the recruitment of vinculin at quiescent cell-cell contacts have not been demonstrated.

**Results:**

We generated vinculin knockdown cells using shRNA specific to vinculin and MDCK epithelial cells. These vinculin-deficient MDCK cells form smaller cell clusters in a suspension than wild-type cells. In wound healing assays, GFP-vinculin accumulated at cell-cell junctions along the wound edge while vinculin-deficient cells displayed a slower wound closure rate compared to vinculin-expressing cells. In the presence of blebbistatin (myosin II inhibitor), vinculin localization at quiescent cell-cell contacts was unaffected while in the presence of jasplakinolide (F-actin stabilizer), vinculin recruitment increased in mature MDCK cell monolayers.

**Conclusion:**

These results demonstrate that vinculin plays an active role at adherens junctions under increased tension at cell-cell contacts where vinculin recruitment occurs in a myosin II activity-dependent manner, whereas vinculin recruitment to the quiescent cell-cell junctions depends on F-actin stabilization.

## Background

Cells experience force and, therefore, need to mechanically respond to stabilize cell junctions with both neighboring cells and the underlying extracellular matrix. Cadherins are the adhesion proteins composing the adherens junctions at cell-cell contacts while cadherin-associated proteins and the actin cytoskeleton provide stability and structural support between neighboring cells. The E-cadherin complex was identified as a mechanosensor at cell-cell contacts where applying force on the extracellular domain of E-cadherin resulted in vinculin-dependent cell stiffening [1]. Vinculin-dependent cell stiffening was also observed at integrin junctions [2,3], suggesting a similar role for vinculin at both force-bearing cell-cell and cell-matrix junctions. Thus, vinculin may be a key protein in generating tension at cell-cell contacts in response to external forces from neighboring cells.

Vinculin was originally identified as a protein associated at the ends of actin fibers terminating at the plasma membrane [4]. Along with F-actin [5,6], additional binding partners to vinculin at focal adhesions include talin [7,8], paxillin [9], α-actinin [10], and phospholipids [11,12]. Vinculin is composed of a head and tail domain that is linked together by a proline-rich linker region and exists in either an open, activated state or a closed, auto-inhibited state where the head and tail domains interact [13-15]. In the open state, previously hidden sites for vinculin binding partners are exposed. Vinculin activation is achieved through interacting with one of several vinculin binding partners [14,16-18].

The association of vinculin with integrins at focal adhesions has been well examined, where vinculin binds to talin and paxillin, two integrin-binding proteins [7-9]. At focal adhesions, vinculin is involved in mechano-coupling between the integrins bound to the underlying extracellular matrix, and the actin cytoskeleton [3,19,20]. In this position, vinculin plays a major role in force-generating processes such as cell migration on a two-dimensional surface [21] and cell invasion in a three-dimensional matrix [22]. Vinculin regulates actomyosin force generation in response to external cues through the vinculin tail domain [3]. Although vinculin at focal adhesions has been well studied, the role of vinculin at cell-cell contacts has not.

In biochemical assays with purified proteins, a direct interaction occurs between the vinculin head domain and the cadherin-associated protein α-catenin [23-25], with the vinculin binding site on α-catenin located between aa 326-509 [14,24]. The additional vinculin interactions with β-catenin [26,27] or myosin VI [28] have also been reported. Interestingly, vinculin recruitment to cell-cell contacts is decreased by the myosin II inhibitor blebbistatin in some epithelial cell lines [1,25,29], thus supporting the role of vinculin in responding to increased tension at cell-cell contacts. An inhibitory region for vinculin binding was identified on α-catenin (aa 510-697) and suggested α-catenin existing in either a closed conformation with the inhibitory domain occluding the vinculin binding site, or in an open conformation under increased tension with the vinculin binding site exposed [25].

MDCK cells are the prototypical polarized epithelial cell model, yet the interaction of vinculin with the E-cadherin complex in MDCK cells is different from other cell lines. This has been attributed to low tension in MDCK cell monolayers under normal conditions possibly due to the unique cadherin distribution of MDCK cell-cell contacts [30], thus resulting in the lack of zonula adherens with vinculin accumulation that is observed in other epithelial cell lines [29]. However, vinculin accumulation increases at adherens junctions lining the wound edge in the MDCK wound-healing model, a process inhibited by blebbistatin [25,29]. Additionally, an α-catenin antibody recognizing a sequence near the vinculin binding site was localized at the junctions along the wound edge, therefore, indicating the availability of vinculin binding sites on α-catenin at cell-cell contacts under increased tension [25]. Also, in HGF-treated highly migratory MDCK cells, increased vinculin recruitment to cell-cell contacts is reversed with the inclusion of blebbistatin, suggesting myosin II-dependence [1]. This indicates that vinculin is recruited under conditions of increased tension to cell-cell contacts in a myosin II activity-dependent manner. Interestingly, in the MDCK cell monolayer with low tension cell-cell contacts, vinculin still localizes to cell-cell contacts. Therefore, additional factors (myosin II-independent) may be recruiting vinculin to sites of cell-cell contacts, but the mechanisms by which vinculin accumulates under these conditions remain unclear.

The aim of this study was to investigate the role of vinculin at force-bearing adherens junctions and its recruitment to sites of cell-cell contact. In this report, using vinculin knockdown and GFP-vinculin expressing cells in wound-healing assays, we demonstrate that vinculin is required for proper wound closure and vinculin-deficiency results in a reduced rate of wound closure. Additionally, we show that recruitment of vinculin to cell-cell contacts is increased with F-actin stabilization.

## Results and Discussion

### Generation of vinculin-deficient cells

**Figure 1:**
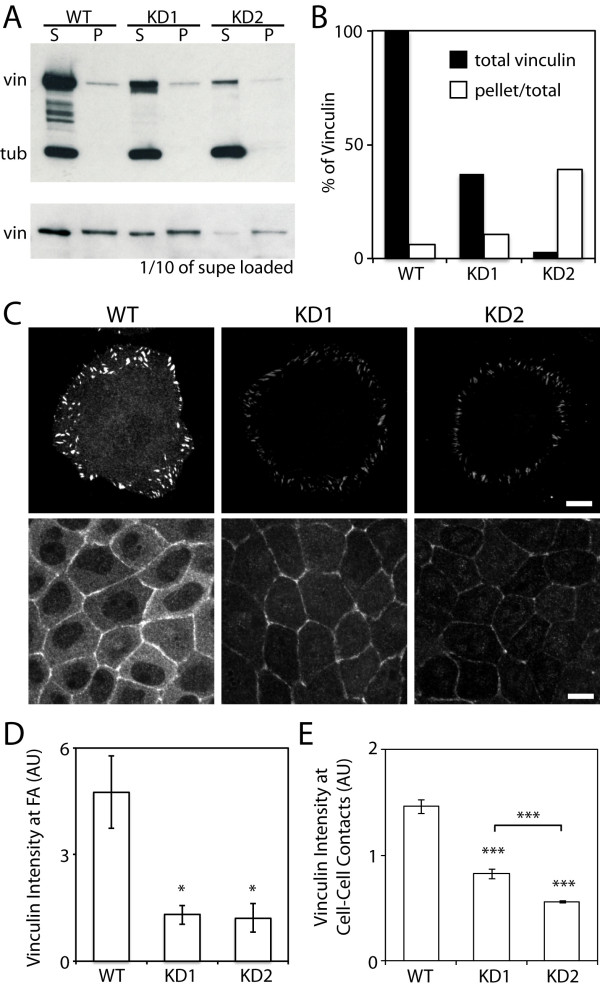
**Characterization of vinculin knock-down cells**. (A) Western blot of detergent soluble and insoluble pool of vinculin in wild-type (WT) and vinculin knockdown (KD) cells. (B) Quantification of total and detergent insoluble pool of vinculin in wild-type and knockdown cells. The fraction of detergent insoluble (pellet) vinculin pool increased in both vinculin knockdown cells. (C) Immuno-fluorescence analysis of vinculin in wild-type and knockdown cells. Although vinculin is depleted from the cytoplasm of knockdown cells, the residual vinculin is present at focal adhesions (top panel) and cell-cell contacts (bottom panel). Images were taken at basal (top panel) and apical (bottom panel) levels. The same exposure and laser power were used to acquire and generate the images. Scale bar 10 μm. (D) Quantification of vinculin at focal adhesions in wild-type and knockdown cells. Immuno-fluorescence intensities and analysis of vinculin at focal adhesions are presented as mean ± standard error of the mean and are compared with control (*P < 0.05). (E) Quantification of vinculin at cell-cell contacts in wild-type and knockdown cells. Immuno-fluorescence intensities and analysis of vinculin at cell-cell contacts are presented as mean ± standard error of the mean and are compared with control (***P < 0.001).

We generated stable cell lines of vinculin knockdown cells using three shRNA target sequences for canine vinculin. For analysis of vinculin in cell-cell adhesion formation, two vinculin knockdown MDCK cell lines were chosen as these subclones represented the intermediate (37%, KD1 from shRNA#1) and least (3%, KD2 from shRNA#2) total vinculin level compared to wild-type (Figure 1B and Additional File 1, Figure S1). Interestingly, relative to the vinculin level in wild-type cells, the detergent insoluble vinculin in knockdown cells were 62% for KD1 and 17% for KD2 (Figure 1A and 1B). Thus, when vinculin level is lowered by shRNA, the detergent soluble, cytoplasmic pool of vinculin is preferentially reduced while less reduction is observed in the detergent insoluble pool. This in turn suggests that residual vinculin is preferentially associated with the actin cytoskeleton in knockdown cells.

To further analyze the relative distribution of vinculin, we immuno-labeled vinculin in wild-type and vinculin knockdown cells. The immuno-labeled vinculin localized to the cytoplasm and focal adhesions in wild-type cells, but significantly less in knockdown cells (Figure 1C and 1D). Preferential vinculin localization to focal adhesions in both knockdown cells (Figure 1C and 1D), albeit less intense than wild-type cells, is consistent with the increased insoluble vinculin pool observed with biochemical analysis (Figure 1A). In a confluent MDCK monolayer, vinculin localized to cell-cell contacts in both wild-type and knockdown cells (Figure 1C), but the vinculin intensity at cell-cell contacts of knockdown cells was much weaker (Figure 1C and 1E). While comparable vinculin levels were present at the focal adhesions between the two knockdown cell lines (Figure 1D), KD2 had significantly less vinculin at cell-cell contacts compared to KD1 (Figure 1E), consistent with KD2 having a lower level of vinculin than KD1 (Figure 1A and 1B). Together, in vinculin knockdown cells, the residual vinculin preferentially localized to focal adhesions and cell-cell contacts while depleting most of the cytoplasmic vinculin pool.

### The vinculin knockdown cells have weaker cell-cell adhesion

**Figure 2:**
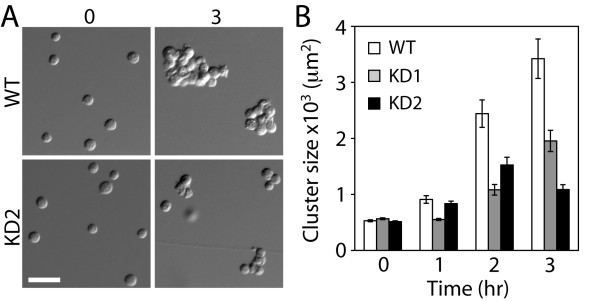
**Vinculin knock-down decreases cell-cell adhesion**. (A) Cell clustering in the hanging drop adhesion assay of wild-type (WT) and knockdown (KD2) cells. Time in hours. Scale bar 50 μm. (B) Quantification of clustering size of wild-type and knockdown (KD1 and KD2) cells. Error bars are standard error of the mean.

Since the residual vinculin in knockdown cells accumulated at cell-cell contacts, albeit much less than wild-type cells, we tested whether the residual vinculin is sufficient to support cell-cell contact formations using the hanging drop adhesion assay. The wild-type and knockdown cells were suspended in a media to allow cell clustering and cell cluster sizes were analyzed over time. The vinculin knockdown cells displayed small cell clusters (Figure 2), suggesting that cell-cell adhesion is weaker compared to wild-type. Comparing the two vinculin knockdown cell lines, both knockdown cells formed smaller cell clusters than wild-type cells, but the more efficient knockdown cells (KD2) had smaller cell clusters than the less efficient knockdown cell line (KD1) after 3 hours (Figure 2). These data suggest that the effects of shRNA are not due to off-target silencing or clonal variation; two different shRNA sequences were used to generate the vinculin-deficient cell lines, yet both cell lines exhibit the knockdown level-dependent, reduced cell clustering, Furthermore, defective cell aggregation observed in the vinculin knockdown cells supports the notion that vinculin is a key contributor to the formation of proper cell-cell adhesion.

### Vinculin is required for efficient wound healing

Despite the reduced vinculin level at cell-cell contacts, vinculin knockdown cells form a cell monolayer without any phenotypic defects (Figure 1C). Since vinculin has been shown to localize to force-bearing sites of wound closing cells, we analyzed collective cell movement during wound healing by creating a single cell-sized opening in a mature monolayer. In this wound healing model, formation and contraction of the actomyosin purse-string surrounding the closing wound generates tension on adherens junctions at cell-cell contacts along the wound edge [29,31]. In addition, vinculin was previously observed as punctate formations at cell-cell contacts along the wound edge, concurrent with increased adherens junctions formation [29].

**Figure 3:**
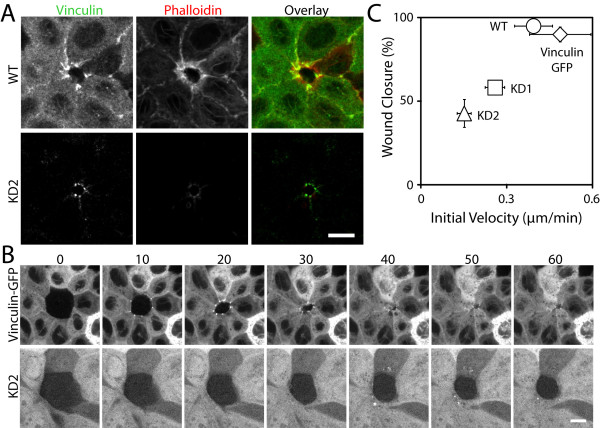
**Vinculin knock-down decreases the rate of wound-healing in MDCK cells**. (A) Following ablation of a single cell in a monolayer, an F-actin purse-string formed along the wound edge with punctate vinculin accumulation at cell-cell contacts in MDCK wild-type (WT) cells. Vinculin knock-down (KD) cells displayed decreased vinculin and F-actin accumulation along the wound edge. The same exposure and laser power were used to acquire and generate the images. Scale bar 10 μm. (B) Time-lapse comparison of a wound-healing assay demonstrates vinculin accumulation at cell-cell contacts along the wound edge along with wound closure for vinculin-expressing (vinculin-GFP) cells and not in KD cells. Stably transfected KD cells are positive for a low level of GFP expression. Time in minutes. Scale bar 10 μm. (C) Comparison of the percentage of wound-closure after 1 hr and the initial velocity of wound closure between WT (n = 8), vinculin-GFP (n = 9), KD1 (n = 7), and KD2 (n = 11) cells. Error bars are standard error of the mean.

In the wound-healing assay, vinculin accumulated at the wound edge in both wild-type and knockdown cells. However, vinculin intensity in the knockdown cells was reduced compared to the wild-type cells (Figure 3A). MDCK cells expressing GFP-vinculin were also used in the wound healing assay to analyze the wound-healing rate of vinculin-expressing versus vinculin-deficient cells. GFP-vinculin rapidly accumulated at adherens junctions along the wound edge (Figure 3B), and the wound rapidly closed within an hour. The GFP-vinculin cells displayed a comparable wound-healing rate to wild-type cells (Figure 3C). In comparison to wild-type and GFP-vinculin expressing MDCK cells, the vinculin-deficient cells had a decreased wound-healing rate and incomplete wound closure after 1 hour (Figure 3B and 3C). The decreased wound-healing rate suggests that the cells along the wound edge are unable to generate sufficient tension through the cytoskeleton at cell-cell contacts to promote wound closure, thus, supporting vinculin as an active participant in force generation at cell-cell contacts.

### Myosin II independent vinculin accumulation at cell-cell contacts

**Figure 4:**
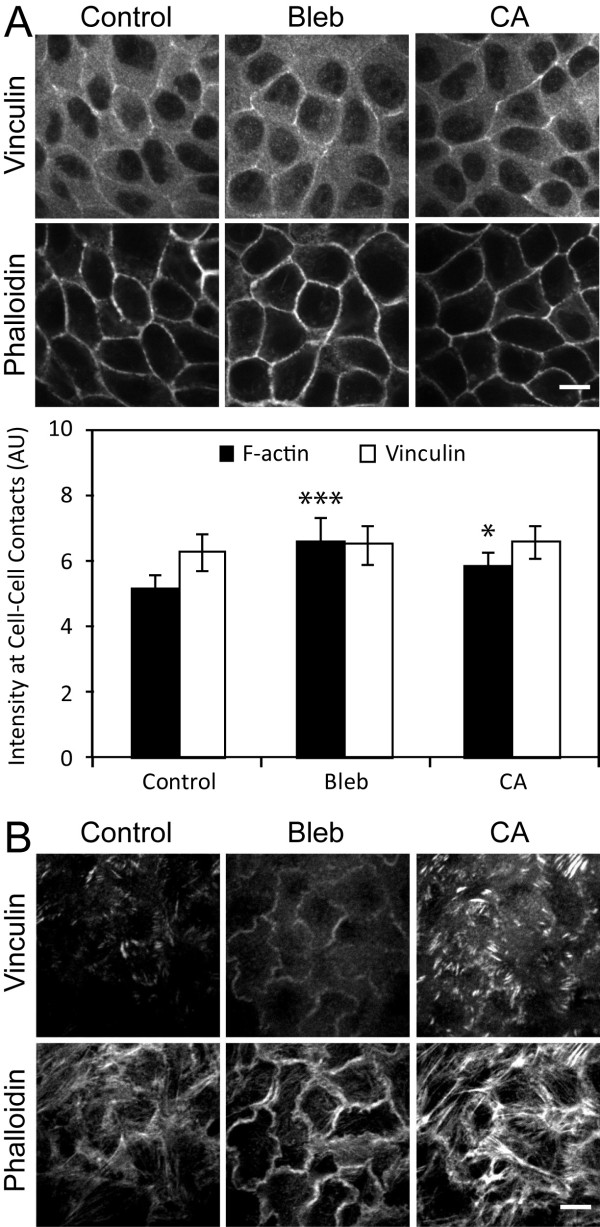
**Myosin II activity and vinculin recruitment to cell-cell contacts of MDCK cells**. (A) Confluent MDCK cell monolayers on collagen-coated coverslips were incubated with media containing 50 μM blebbistatin (Bleb), 10 nM calyculin-A (CA), or media alone (Control) for 1 hr. Cells were fixed and immunostained for vinculin or F-actin (phalloidin) at cell-cell contacts. The same exposure and laser power were used to acquire and generate the images. Scale bar 10 μm. Data of immuno-fluorescence intensities and analysis of F-actin and vinculin at cell-cell contacts are presented as mean ± standard deviation and are compared with control (n = 10, *P < 0.05, ***P < 0.001). (B) Immuno-fluorescence of vinculin and F-actin at basal level of confluent MDCK cells. Scale bar 10 μm.

Vinculin localizes along the length of cell-cell contacts in mature MDCK cell monolayers [1,25] (Figure 1). Since blebbistatin dissociates vinculin from cell-cell contacts in other epithelial cell lines, MCF7 [28] and MTD-1A [29], vinculin localization is thought to be myosin II activity-dependent. Unlike other cell types, however, the vinculin distribution at cell-cell contacts in normal MDCK cell monolayers is not affected by blebbistatin treatment (Figure 4A), whereas the vinculin intensity at focal adhesions is reduced (Figure 4B). This myosin II activity-independent vinculin localization in MDCK cells has been attributed to the lack of tension at cell-cell contacts in normal MDCK cells [29], albeit without direct analysis of tension.

Calyculin-A is a serine/threonine phosphatase inhibitor that inhibits Myosin Light Chain (MLC) phosphatase. The inhibition of MLC phosphatase is thought to induce myosin II activity by minimizing the de-phosphorylation of MLC, thus myosin II remains in a phosphorylated and active state. In the presence of calyculin-A, numerous actin bundles formed at the basal surface (Figure 4B), a sign of increased myosin II-dependent contractility by calyculin A. Interestingly, the intensity of vinculin at cell-cell contacts was unchanged (Figure 4A). This observation suggests that a mere increase in myosin II activation does not recruit vinculin to cell-cell contacts of MDCK cells. Although myosin II deactivation is sufficient for the vinculin dissociation from cell-cell contacts in some cells, the vinculin recruitment to cell-cell contacts may require additional factors.

### Actin stabilization recruits vinculin to cell-cell contacts

**Figure 5:**
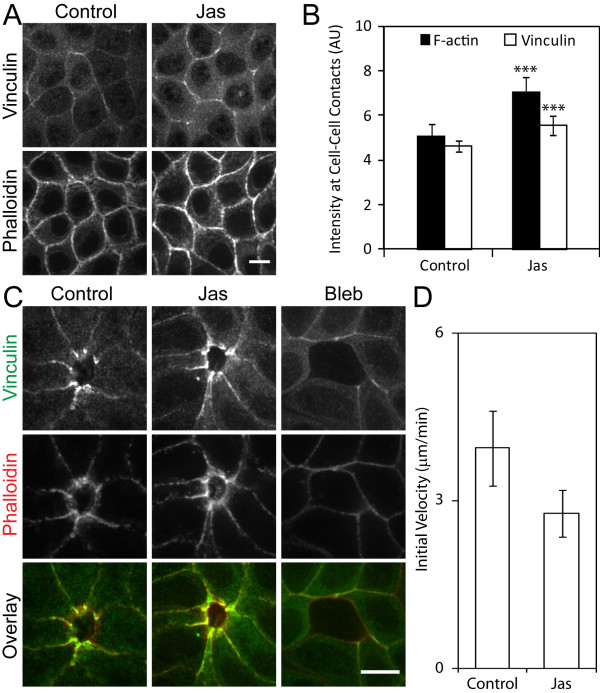
**Vinculin recruitment to cell-cell contacts occurs with F-actin stabilization in MDCK cells**. (A) Confluent MDCK cell monolayers on collagen-coated coverslips were incubated with media containing 200 nM jasplakinolide (Jas) or media alone (Control), fixed, and immuno-stained for vinculin and F-actin (phalloidin) at cell-cell contacts. The same exposure and laser power were used to acquire and generate the images. Scale bar 10 μm. (B) Data of immuno-fluorescence intensities and analysis of F-actin and vinculin at cell-cell contacts are presented as mean ± standard deviation and are compared with control (n = 10, ***P < 0.001). (C) Single cell ablations of confluent MDCK wild-type cell monolayers were performed following treatment with media containing 200 nM Jas, 50 μM blebbistatin (Bleb), or media alone (Control). Vinculin and actin accumulated along the wound edge in Jas-treated and control cells, but was reduced with Bleb treatment. Scale bar 10 μm. (D) Comparison of the initial velocity of wound-closure between control (n = 8) and Jas-treated (n = 7) cells. Error bars are standard error of the mean.

One factor that may be involved in recruiting vinculin to cell-cell contacts is the amount or dynamic state of F-actin, a binding partner to which vinculin contains F-actin binding domains [5,6]. To determine whether vinculin recruitment to cell-cell contacts depends on F-actin stabilization, cells were treated with jasplakinolide. In confluent MDCK cell monolayers, jasplakinolide addition lowers the turnover of the actin network at cell-cell contacts [32]. The addition of jasplakinolide increased both the phalloidin and vinculin intensity at cell-cell contacts (Figure 5A and 5B). At the basal surface, jasplakinolide also increased vinculin recruitment to focal adhesions and actin bundle formation (Additional file 1, Figure S2). These results suggest that F-actin formation or stabilization is an additional factor required for vinculin accumulation at cell-cell contacts in MDCK cells. Interestingly, an increase in F-actin intensity at cell-cell contacts was observed with blebbistatin-treated MDCK cells (Figure 4A), yet the vinculin level at cell-cell contacts was unaffected, suggesting that the stabilization of F-actin, not the amount of F-actin, is required for vinculin recruitment at cell-cell contacts.

To test whether stabilizing F-actin affects vinculin recruitment to cell-cell contacts under tension, jasplakinolide treatment was included in the MDCK wound-healing assay. In the presence of jasplakinolide, vinculin accumulated at cell-cell contacts along the wound edge similarly to control (Figure 5C). Although there was no significant difference in initial wound-healing rates between control and jasplakinolide-treated cells, a trend towards jasplakinolide decreasing initial velocity was observed (Figure 5D). This suggests that actin stabilization reduces the wound closure rate, and that actin dynamics is required for efficient wound closure.

On the other hand, vinculin accumulation was either reduced or absent along the wound edge in blebbistatin-treated cells (Figure 5C), likely due to the lack of contractile actin network. Consistent with a previous study [29], these cells failed to close the wound, suggesting that myosin II activity is required for wound closure. Since jasplakinolide treatment did not change vinculin recruitment to the wound edge, the vinculin recruitment is solely dependent on myosin II activity. This is in contrast to a mature cell monolayer, where vinculin recruitment requires F-actin stabilization.

Our data suggests the existence of two vinculin pools at the sites of cell-cell adhesion: myosin II dependent and independent pools. In MDCK cells, the myosin II dependent pool is revealed only when cell-cell contacts are under high tension (e.g. force-bearing contacts during wound healing, Figure 5C), while at quiescent cell-cell contacts, vinculin recruitment is enhanced by pharmacologically stabilized F-actin (Figure 5A and 5B). Note that, at quiescent cell-cell contacts, both blebbistatin (Figure 4A) and jasplakinolide treatments (Figure 5A and 5B) increased F-actin intensity, but, only F-actin stabilization with jasplakinolide increased vinculin recruitment at cell-cell contacts, suggesting that the F-actin level does not always correlate with the vinculin recruitment. The recruitment of the contractility-dependent vinculin pool is likely mediated by force-sensitive conformational change of α-catenin, which in turn exposes a previously masked vinculin binding site [25]. Since force-bearing sites of wound edge cells contain VASP recruited by zyxin to promote actin polymerization [33], vinculin may also facilitate actin dynamics, possibly through its barbed-end nucleation activity [34], necessary for the force transmission.

At quiescent cell-cell contacts, jasplakinolide-induced actin stabilization promotes vinculin accumulation. The actin side-binding/bundling activity of vinculin may be important for the maintenance of stable actin network at cell-cell contacts. A low vinculin concentration at MDCK cell-cell contacts is consistent with unstable actin dynamics observed at mature cell-cell contacts [32]. The physiological mechanism of actin stabilization remains unclear, but α-catenin dimers have been shown to interact with actin filaments to promote actin bundle formation [35]. Therefore, both α-catenin and vinculin may work together to maintain the stable actin network at mature cell-cell junctions. Furthermore, in Drosophila embryos, two distinct actin dynamics are present to regulate cadherin mobility [36]. These two distinct vinculin pools may be critical for the key divergence of actin function and may be a fundamental feature of actin regulation at cell-cell contacts.

## Conclusions

The major findings of this study are that vinculin is required for both proper cell-cell adhesion and wound closure through contraction of the actomyosin purse-string. Additionally, vinculin recruitment to quiescent cell-cell contacts occurs with F-actin stabilization in MDCK cells. These results demonstrate vinculin to actively participate in generating force at cell-cell contacts and that vinculin localization at cell-cell contacts may occur in both a myosin II activity-dependent and independent manner.

## Methods

### Cell lines and reagents

MDCK GII cells were cultured in Dulbecco's modified Eagle's medium (low glucose) supplemented with 10% fetal bovine serum, penicillin, streptomycin, and kanamycin. MDCK cells stably expressing GFP-vinculin was previously described [32]. Primary antibodies used were mouse monoclonal IgGs against vinculin (clone hVIN-1, Sigma), E-cadherin (clone 36, BD Biosciences), and tubulin (clone DM1A, Sigma). Myosin II activity was perturbed with 50 μM blebbistatin (Calbiochem) or 10 nM calyculin A (Calbiochem) while 200 nM jasplakinolide (Molecular Probes) was used to stabilize F-actin.

### shRNA

Canine vinculin-specific oligonucleotide sequences (shRNA1: 5'-TGAAGAGAGATATGCCACC-3', shRNA2: 5'-AAGGCAAGATTGAGCAAGC-3', or shRNA3: 5'-CAAGCTGCTTACGAACACT-3') were inserted into the pSUPER.gfp/neo expression vector (Oligoengine), and transfected into MDCK cells using Lipofectamine 2000 (Invitrogen). Stable vinculin knockdown cells were subcloned and screened for low vinculin levels using Western blot (see an example blot in Figure S1). The total number of subclones was 25 (shRNA1), 19 (shRNA2), and 16 (shRNA3) clones. Stable cell lines were maintained with 100 μg/ml G418 treatment.

### Microscopy and live-cell imaging

Cells were imaged in a 37°C temperature-controlled chamber using a Zeiss AxioObserver equipped with a Yokogawa spinning disk confocal system, 40× and 63× objectives, 488 and 561 nm solid-state lasers, and a Cool SNAP HQ camera. The microscope system was controlled by Slidebook software (Intelligent Imaging Innovations).

### Wound-healing assay

A photo-ablation system (Intelligent Imaging Innovation) was used to ablate a single cell within a cell monolayer. The subsequent wound area was imaged every minute for 1 hr and the area of the wound opening was quantified using ImageJ. All images were acquired and generated using the same exposure, laser power, and gain. Wound size measurements within the first 10 min following cell ablation were used to analyze the initial velocity of wound closure.

### Hanging drop assay

Cells were suspended at a density of 2.5 × 10^5 ^cells/ml medium. 25 μl of cell suspension was seeded onto glass-bottom dishes, inverted upside-down, and incubated at 37°C. Cell suspensions were then triturated through a pipette tip 30 times and the cluster sizes were quantified using ImageJ.

### Immuno-fluorescence and image analysis

MDCK cells were seeded onto collagen-coated coverslips. Cells were fixed in 3% para-formaldehyde containing 0.3% TritonX-100 for 10 min and blocked with 1% BSA containing 0.3% TritonX-100. Following primary antibody incubation, immuno-labeled cells were detected with AlexaFluor 488-conjugated secondary antibodies (Molecular Probes). Cells were counter-stained with AlexaFluor 568-phalloidin (Molecular Probes) to detect F-actin. Fluorescence intensities were quantified using ImageJ. All images were acquired and generated using the same exposure and laser power. Fluorescence intensities were measured at cell-cell junctions by obtaining selection areas specific to F-actin staining at cell-cell contacts. The same selection areas obtained with F-actin images were overlaid onto corresponding vinculin images to obtain vinculin intensities along the cell-cell contacts (example images shown in Additional file 1, Figure S3).

### Statistical analyses

Results comparing two groups were analyzed using a two-tailed, unpaired Student's *t*-test assuming equal variance. Results comparing three groups were analyzed using a one-way ANOVA and significance between groups was determined using the Bonferroni correction. Results were considered significant when P < 0.05. Data was analyzed using Microsoft Excel.

## List of abbreviations

GFP: green fluorescent protein; MDCK: Madin-Darby canine kidney; MLC: myosin light chain.

## Authors' contributions

GS carried out vinculin immunostaining following treatments, wound healing analysis in the presence of treatments, and drafted the manuscript. TT carried out the wound healing assay characterization of vinculin knock-down cells. WS carried out the cell adhesion assay. SY conceived the experiments, carried out the characterization of vinculin knockdown cells and wound healing assay, and drafted the manuscript. All authors read and approved the manuscript.

## Supplementary Material

Additional file 1 **Figure S1. Stable subclones of vinculin knockdown cells**. Western blot of wild-type (WT) and vinculin knockdown (KD) cells using vinculin (vin) and tubulin (tub) antibodies. Three independent shRNA sequences were used (see Methods for detail). **Figure S2. Vinculin recruitment to focal adhesions occurs with F-actin stabilization in MDCK cells**. Confluent MDCK cell monolayers on collagen-coated coverslips were incubated with media containing 200 nM jasplakinolide (Jas) or media alone (Control), fixed, and immune-stained for vinculin and F-actin (phalloidin) at focal adhesions. The same exposure and laser power were used to acquire and generate the images. Scale bar 10 μm. **Figure S3. Fluorescence intensity analysis of vinculin and actin at cell-cell contacts**. Selection areas for fluorescence intensity for analysis were generated using F-actin (phalloidin) staining at cell-cell contacts. Selection areas were then overlaid onto vinculin images to measure vinculin fluorescence intensities at cell-cell contacts. Click here for file

## References

[B1] le Duc Q Shi Q Blonk I Sonnenberg A Wang N Leckband D de Rooij J Vinculin potentiates E-cadherin mechanosensing and is recruited to actin-anchored sites within adherens junctions in a myosin II-dependent manner J Cell Biol 189 7 1107 1115 10.1083/jcb.201001149 PMC2894457 20584916

[B2] Alenghat FJ Fabry B Tsai KY Goldmann WH Ingber DE Analysis of cell mechanics in single vinculin-deficient cells using a magnetic tweezer Biochem Biophys Res Commun 2000 277 1 93 99 10.1006/bbrc.2000.3636 11027646

[B3] Mierke CT Kollmannsberger P Zitterbart DP Smith J Fabry B Goldmann WH Mechano-coupling and regulation of contractility by the vinculin tail domain Biophys J 2008 94 2 661 670 10.1529/biophysj.107.108472 17890382 PMC2481521

[B4] Geiger B A 130K protein from chicken gizzard: its localization at the termini of microfilament bundles in cultured chicken cells Cell 1979 18 1 193 205 10.1016/0092-8674(79)90368-4 574428

[B5] Menkel AR Kroemker M Bubeck P Ronsiek M Nikolai G Jockusch BM Characterization of an F-actin-binding domain in the cytoskeletal protein vinculin J Cell Biol 1994 126 5 1231 1240 10.1083/jcb.126.5.1231 8063860 PMC2120156

[B6] Huttelmaier S Bubeck P Rudiger M Jockusch BM Characterization of two F-actin-binding and oligomerization sites in the cell-contact protein vinculin Eur J Biochem 1997 247 3 1136 1142 10.1111/j.1432-1033.1997.01136.x 9288940

[B7] Burridge K Mangeat P An interaction between vinculin and talin Nature 1984 308 5961 744 746 10.1038/308744a0 6425696

[B8] Bass MD Patel B Barsukov IG Fillingham IJ Mason R Smith BJ Bagshaw CR Critchley DR Further characterization of the interaction between the cytoskeletal proteins talin and vinculin Biochem J 2002 362 Pt 3 761 768 11879206 10.1042/0264-6021:3620761 PMC1222443

[B9] Wood CK Turner CE Jackson P Critchley DR Characterisation of the paxillin-binding site and the C-terminal focal adhesion targeting sequence in vinculin J Cell Sci 1994 107 Pt 2 709 717 8207093

[B10] McGregor A Blanchard AD Rowe AJ Critchley DR Identification of the vinculin-binding site in the cytoskeletal protein alpha-actinin Biochem J 1994 301 Pt 1 225 233 8037676 10.1042/bj3010225 PMC1137166

[B11] Weekes J Barry ST Critchley DR Acidic phospholipids inhibit the intramolecular association between the N- and C-terminal regions of vinculin, exposing actin-binding and protein kinase C phosphorylation sites Biochem J 1996 314 Pt 3 827 832 8615776 10.1042/bj3140827 PMC1217131

[B12] Johnson RP Niggli V Durrer P Craig SW A conserved motif in the tail domain of vinculin mediates association with and insertion into acidic phospholipid bilayers Biochemistry 1998 37 28 10211 10222 10.1021/bi9727242 9665728

[B13] Johnson RP Craig SW F-actin binding site masked by the intramolecular association of vinculin head and tail domains Nature 1995 373 6511 261 264 10.1038/373261a0 7816144

[B14] Bakolitsa C Cohen DM Bankston LA Bobkov AA Cadwell GW Jennings L Critchley DR Craig SW Liddington RC Structural basis for vinculin activation at sites of cell adhesion Nature 2004 430 6999 583 586 10.1038/nature02610 15195105

[B15] Borgon RA Vonrhein C Bricogne G Bois PR Izard T Crystal structure of human vinculin Structure 2004 12 7 1189 1197 10.1016/j.str.2004.05.009 15242595

[B16] Izard T Evans G Borgon RA Rush CL Bricogne G Bois PR Vinculin activation by talin through helical bundle conversion Nature 2004 427 6970 171 175 10.1038/nature02281 14702644

[B17] Izard T Vonrhein C Structural basis for amplifying vinculin activation by talin J Biol Chem 2004 279 26 27667 27678 10.1074/jbc.M403076200 15070891

[B18] Bois PR O'Hara BP Nietlispach D Kirkpatrick J Izard T The vinculin binding sites of talin and alpha-actinin are sufficient to activate vinculin J Biol Chem 2006 281 11 7228 7236 10.1074/jbc.M510397200 16407299

[B19] Ezzell RM Goldmann WH Wang N Parashurama N Ingber DE Vinculin promotes cell spreading by mechanically coupling integrins to the cytoskeleton Exp Cell Res 1997 231 1 14 26 10.1006/excr.1996.3451 9056408

[B20] Humphries JD Wang P Streuli C Geiger B Humphries MJ Ballestrem C Vinculin controls focal adhesion formation by direct interactions with talin and actin J Cell Biol 2007 179 5 1043 1057 10.1083/jcb.200703036 18056416 PMC2099183

[B21] Goldmann WH Schindl M Cardozo TJ Ezzell RM Motility of vinculin-deficient F9 embryonic carcinoma cells analyzed by video, laser confocal, and reflection interference contrast microscopy Exp Cell Res 1995 221 2 311 319 10.1006/excr.1995.1380 7493629

[B22] Mierke CT Kollmannsberger P Zitterbart DP Diez G Koch TM Marg S Ziegler WH Goldmann WH Fabry B Vinculin facilitates cell invasion into three-dimensional collagen matrices J Biol Chem 285 17 13121 13130 10.1074/jbc.M109.087171 PMC2857131 20181946

[B23] Weiss EE Kroemker M Rudiger AH Jockusch BM Rudiger M Vinculin is part of the cadherin-catenin junctional complex: complex formation between alpha-catenin and vinculin J Cell Biol 1998 141 3 755 764 10.1083/jcb.141.3.755 9566974 PMC2132754

[B24] Watabe-Uchida M Uchida N Imamura Y Nagafuchi A Fujimoto K Uemura T Vermeulen S van Roy F Adamson ED Takeichi M alpha-Catenin-vinculin interaction functions to organize the apical junctional complex in epithelial cells J Cell Biol 1998 142 3 847 857 10.1083/jcb.142.3.847 9700171 PMC2148175

[B25] Yonemura S Wada Y Watanabe T Nagafuchi A Shibata M alpha-Catenin as a tension transducer that induces adherens junction development Nat Cell Biol 12 6 533 542 10.1038/ncb2055 20453849

[B26] Hazan RB Kang L Roe S Borgen PI Rimm DL Vinculin is associated with the E-cadherin adhesion complex J Biol Chem 1997 272 51 32448 32453 10.1074/jbc.272.51.32448 9405455

[B27] Peng X Cuff LE Lawton CD DeMali KA Vinculin regulates cell-surface E-cadherin expression by binding to beta-catenin J Cell Sci 123 Pt 4 567 577 10.1242/jcs.056432 PMC2818194 20086044

[B28] Maddugoda MP Crampton MS Shewan AM Yap AS Myosin VI and vinculin cooperate during the morphogenesis of cadherin cell cell contacts in mammalian epithelial cells J Cell Biol 2007 178 3 529 540 10.1083/jcb.200612042 17664339 PMC2064848

[B29] Miyake Y Inoue N Nishimura K Kinoshita N Hosoya H Yonemura S Actomyosin tension is required for correct recruitment of adherens junction components and zonula occludens formation Exp Cell Res 2006 312 9 1637 1650 10.1016/j.yexcr.2006.01.031 16519885

[B30] Nathke IS Hinck L Swedlow JR Papkoff J Nelson WJ Defining interactions and distributions of cadherin and catenin complexes in polarized epithelial cells J Cell Biol 1994 125 6 1341 1352 10.1083/jcb.125.6.1341 8207062 PMC2290918

[B31] Rosenblatt J Raff MC Cramer LP An epithelial cell destined for apoptosis signals its neighbors to extrude it by an actin- and myosin-dependent mechanism Curr Biol 2001 11 23 1847 1857 10.1016/S0960-9822(01)00587-5 11728307

[B32] Yamada S Pokutta S Drees F Weis WI Nelson WJ Deconstructing the cadherin-catenin-actin complex Cell 2005 123 5 889 901 10.1016/j.cell.2005.09.020 16325582 PMC3368712

[B33] Nguyen TN Uemura A Shih W Yamada S Zyxin-mediated actin assembly is required for efficient wound closure J Biol Chem 285 46 35439 35445 10.1074/jbc.M110.119487 PMC2975167 20801875

[B34] Le Clainche C Dwivedi SP Didry D Carlier MF Vinculin is a dually regulated actin filament barbed end-capping and side-binding protein J Biol Chem 285 30 23420 23432 10.1074/jbc.M110.102830 PMC2906333 20484056

[B35] Drees F Pokutta S Yamada S Nelson WJ Weis WI Alpha-catenin is a molecular switch that binds E-cadherin-beta-catenin and regulates actin-filament assembly Cell 2005 123 5 903 915 10.1016/j.cell.2005.09.021 16325583 PMC3369825

[B36] Cavey M Rauzi M Lenne PF Lecuit T A two-tiered mechanism for stabilization and immobilization of E-cadherin Nature 2008 453 7196 751 756 10.1038/nature06953 18480755

