# Supplementary material for: Myosin II activity dependent and independent vinculin recruitment to the sites of E-cadherin-mediated cell-cell adhesion
Source: BMC Cell Biol. 2011 Nov 3;12:48. doi: 10.1186/1471-2121-12-48 (PMC3215179; doi:10.1186/1471-2121-12-48)
Supplement: Additional file 1 — Figure S1. Stable subclones of vinculin knockdown cells. Western blot of wild-type (WT) and vinculin knockdown (KD) cells using vinculin (vin) and tubulin (tub) antibodies. Three independent shRNA sequences were used (see Methods for detail). Figure S2. Vinculin recruitment to focal adhesions occurs with F-actin stabilization in MDCK cells. Confluent MDCK cell monolayers on collagen-coated coverslips were incubated with media containing 200 nM jasplakinolide (Jas) or media alone (Control), fixed, and immune-stained for vinculin and F-actin (phalloidin) at focal adhesions. The same exposure and laser power were used to acquire and generate the images. Scale bar 10 μm. Figure S3. Fluorescence intensity analysis of vinculin and actin at cell-cell contacts. Selection areas for fluorescence intensity for analysis were generated using F-actin (phalloidin) staining at cell-cell contacts. Selection areas were then overlaid onto vinculin images to measure vinculin fluorescence intensities at cell-cell contacts. [file 1471-2121-12-48-S1.PDF]

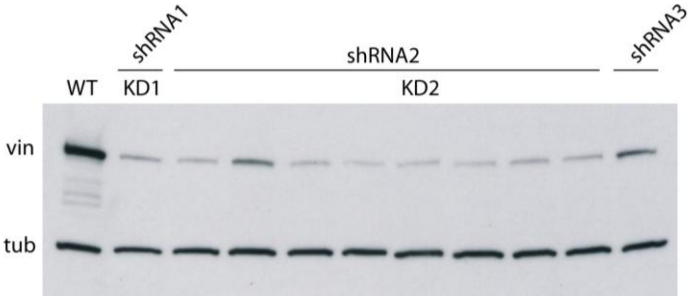

Figure S1. Stable subclones of vinculin knockdown cells. Western blot of wild-type (WT) and vinculin knockdown (KD) cells using vinculin (vin) and tubulin (tub) antibodies. Three independent shRNA sequences were used (see Methods for detail).

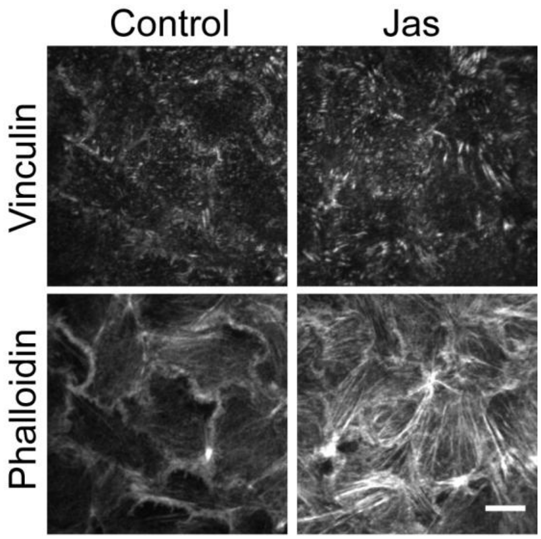

Figure S2. Vinculin recruitment to focal adhesions occurs with F-actin stabilization in MDCK cells. Confluent MDCK cell monolayers on collagen-coated coverslips were incubated with media containing 200 nM jasplakinolide (Jas) or media alone (Control), fixed, and immune-stained for vinculin and F-actin (phalloidin) at focal adhesions. The same exposure and laser power were used to acquire and generate the images. Scale bar 10 µm.

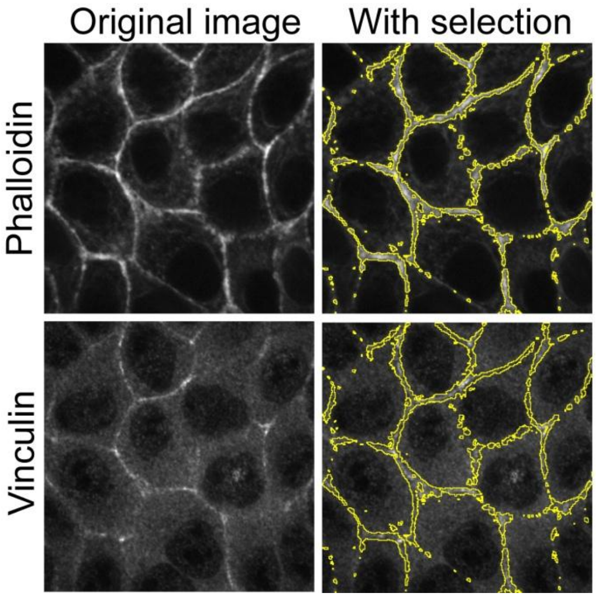

Figure S3. Fluorescence intensity analysis of vinculin and actin at cell-cell contacts. Selection areas for fluorescence intensity for analysis were generated using F-actin (phalloidin) staining at cell-cell contacts. Selection areas were then overlaid onto vinculin images to measure vinculin fluorescence intensities at cell-cell contacts.
